# A portable analog front-end system for label-free sensing of proteins using nanowell array impedance sensors

**DOI:** 10.1038/s41598-022-23286-7

**Published:** 2022-11-22

**Authors:** Muhammad Tayyab, Pengfei Xie, Muhammad Ahsan Sami, Hassan Raji, Zhongtian Lin, Zhuolun Meng, Seyed Reza Mahmoodi, Mehdi Javanmard

**Affiliations:** Department of Electrical and Computer Engineering, Rutgers University, New Brunswick, 08901 USA

**Keywords:** Electrical and electronic engineering, Diagnostic markers, Predictive markers, Prognostic markers, Diagnostic markers, Predictive markers, Prognostic markers

## Abstract

Proteins are useful biomarkers for a wide range of applications such as cancer detection, discovery of vaccines, and determining exposure to viruses and pathogens. Here, we present a low-noise front-end analog circuit interface towards development of a portable readout system for the label-free sensing of proteins using Nanowell array impedance sensing with a form factor of approximately 35*cm*^2^. The electronic interface consists of a low-noise lock-in amplifier enabling reliable detection of changes in impedance as low as 0.1% and thus detection of proteins down to the picoMolar level. The sensitivity of our system is comparable to that of a commercial bench-top impedance spectroscope when using the same sensors. The aim of this work is to demonstrate the potential of using impedance sensing as a portable, low-cost, and reliable method of detecting proteins, thus inching us closer to a Point-of-Care (POC) personalized health monitoring system. We have demonstrated the utility of our system to detect antibodies at various concentrations and protein (45 pM IL-6) in PBS, however, our system has the capability to be used for assaying various biomarkers including proteins, cytokines, virus molecules and antibodies in a portable setting.

## Introduction

The detection and analysis of proteins, cytokines, and nucleic acids play a key role in a wide range of applications; a few of these include early detection of cancer^1^, discovery of vaccines^2^, and even serological assays for determining exposure to deadly infectious diseases such as SARS-CoV-2^3,4^. Affinity based biosensors such as protein array technologies are a useful platform for carrying out proteomic analysis^5,6^. The use of protein microarrays has been demonstrated for the study and analysis of genes^7^, transcriptomes^8,9^, proteins^10^, and antibodies^11^. Affinity based biosensors are divided into two main categories, namely label-based and label-free biosensors. Label-based sensors are sensors that typically have a fluorescent label to identify the biomarker of interest. Label-based devices traditionally require bulky and expensive microscopes and imaging equipment. Devices employing label-free detection of biomarkers have numerous advantages over their label-based counterparts when it comes to portability and commercialization^12^. Although there has been progress in miniaturizing the label-based sensors by using smartphone cameras instead of bulky microscopes^13^, the label-free techniques eliminate the need for various reagents required for labelling, thus reducing the complexity and cost at the expense of a slight decrease in the detection limit and specificity. In addition, the elimination of the need for preparing and using label molecules abolishes the requirement of specially trained staff, which is generally required for handling the regents and carrying out diagnostics.

Label-free technologies for the detection of proteins have made significant advancements in the last decade and have shown tremendous potential to be used as Point-of-Care devices in the literature^14–16^. Electrochemical biosensing technologies have the benefit of inherently having relatively high sensitivity and the ability to be used with miniaturized hardware thus having a smaller form factor than other technologies such as mass spectrometry or optical biosensing^17^. Electrochemical biosensors can be broadly classified into potentiometric, amperometric, and impedance biosensors based on the electrical parameter being measured. Potentiometric biosensors employ ion-sensitive field effect transistors (ISFETS) and ion selective electrodes for measuring a change in the electric potential due to accumulation of ions as a result of an enzymatic reaction^18^. Wang et al. have demonstrated the use of potentiometric sensors for the detection of carcinoembryonic antigen (CEA) with a sensitivity of 2.5 ng/ml^19^. Amperometric biosensors measure minuscule changes in the electrical current that take place due to redox reactions. The working electrode in such sensors is usually made of an inert material covered with a biorecognition element^20^.

Recently, Fe_3_O_4_ nanoparticles on graphene oxide sheets were used for ultra-sensitive amperometric detection of Prostate Specific Antigen (PSA) with a Limit of Detection (LOD) of 15 fg/ml^21^. Impedance based biosensors detect a change in impedance due to binding of target molecules to the probe molecules immobilized on the surface of the sensor^22^. Mok et al. presented a novel two-chamber impedance sensing microfluidic platform for the detection of proteins^23^. The use of bioactivated microfluidic channels for electrical detection of protein biomarkers using impedance sensing has also been investigated^24^. Recently, picomolar level detection of protein biomarkers using impedance sensing was demonstrated based on the electronic sizing of bead aggregates^25^.

A fully integrated wearable impedance cytometer with an online smartphone readout has been demonstrated a little while back^26^. Recently, a handheld portable platform using disposable nanopore strips has also been presented for the detection of HIV antibody levels in saliva^27^. In another paper^28^, the authors presented a dielectrophoretic-impedance based method for the detection and analysis of proteins, specifically Bovine Serum Albumin (BSA). Authors in another article^29^ showcase an electrochemical impedance-based sensor employing graphene-carbon nanotubes composite deposited on a glassy carbon electrode to detect the protein antigen. A universal antibody-modified nanocrystalline boron-doped diamond biosensor for direct detection of protein and viral particles at very low concentrations was discussed^30^. Researchers described a new paper based electrochemical impedance biosensor for label-free detection and quantification of human interferon-gamma (IFN-γ), a biomarker which plays an important role in tuberculosis susceptibility^31^. Another microfluidic device, utilizing bead-based capture chamber technology, for the quantification of IL-6 in human serum samples for sepsis stratification was presented^32^. Furthermore, in other studies^33,34^, researchers present electrochemical biosensors for the detection of IgG anti-Trypanosoma cruzi antibodies and IgG antibodies to Helicobacter pylori in human serum samples respectively. Table 1 presents a comparison of systems that detect proteins electrically. Although significant leaps in the prototyping and development of microfluidic electrochemical biosensors have been made in the laboratory and academic environment within the last decade, the translation of most of these devices to a Point-of-Care setting has been limited by practical constraints and challenges such as portability, cost, integration, and regulatory affairs^35^. Additionally, the use of electrochemical biosensors for complex physiological samples such as blood, serum, and saliva is challenging since high salt concentrations in these samples reduce the double layer thickness and result in charge screening. Therefore, an electrical system needs to be highly sensitive, portable, and must have the ability to work with biological specimens with high salt concentrations for potential use in a POC setting.

**Table 1:** A tabular comparison of the sample preparation, sample volume, sample to answer time, and portability of sensor systems.

Reference No	Sample preparation	Sample volume	Sample to answer time	Portability
21	Extensive	> 10 µl	> 60 min	No
23	Moderate	< 5 µl	> 60 min	No
24	Moderate	Not Available	> 45 min	No
25	Moderate	Not Available	> 60 min	No
**27**	**Moderate**	**Not Available**	** < 10 min**	**Yes**
28	Moderate	5 µl	< 10 min	No
29	Moderate	10 µl	> 10 min	No
30	Minimal	40 µl	> 30 min	No
31	Moderate	> 100 µl	> 30 min	No
32	Extensive	10 µl	> 3 h	No
33	Extensive	25 µl	< 30 min	No
34	Extensive	12.5 µl	< 30 min	No
36	Minimal	< 10 µl	< 10 min	No
37	Minimal	< 10 µl	> 30 min	No
**Proposed System**	**One-step**	** < 10 µl**	** < 10 min**	**Yes**

Significant values are in bold.

Previously, we have demonstrated the detection of cytokines in serum at femto-molar levels with a dynamic range of 2 orders of magnitude using our nanowell array impedance sensors with a commercial benchtop lock-in amplifier (Zurich Instruments, HF2IS)^36,37^. Although the sensor has the advantage of performing well in a high salt concentration environment which is the case in physiological samples such as blood and serum, the use of the commercial lock-in amplifier with the sensor in a field setting poses challenges to commercialization due to its cost and portability and thus presents an obstacle for its use as a Point-of-Care device. Here, we present the design of a portable, low-noise electronic readout system to be used in conjunction with the nanowell array impedance sensor. We have included a head-to-head comparison of our custom readout system with the commercial lock-in amplifier for the detection of proteins at picomolar levels in Table S1. We have carried out a comprehensive noise analysis of the system and demonstrated its ability to quantify proteins by carrying out experiments. In these experiments we adsorb probe antibodies to the sensor surface and detect the change in impedance due to the binding of the protein with probe molecules. We also present the ability of the system to detect antibodies at various concentrations when the probe molecules are adsorbed to the sensor surface.

## Methods

### The analog front-end system architecture

**Figure 1:**
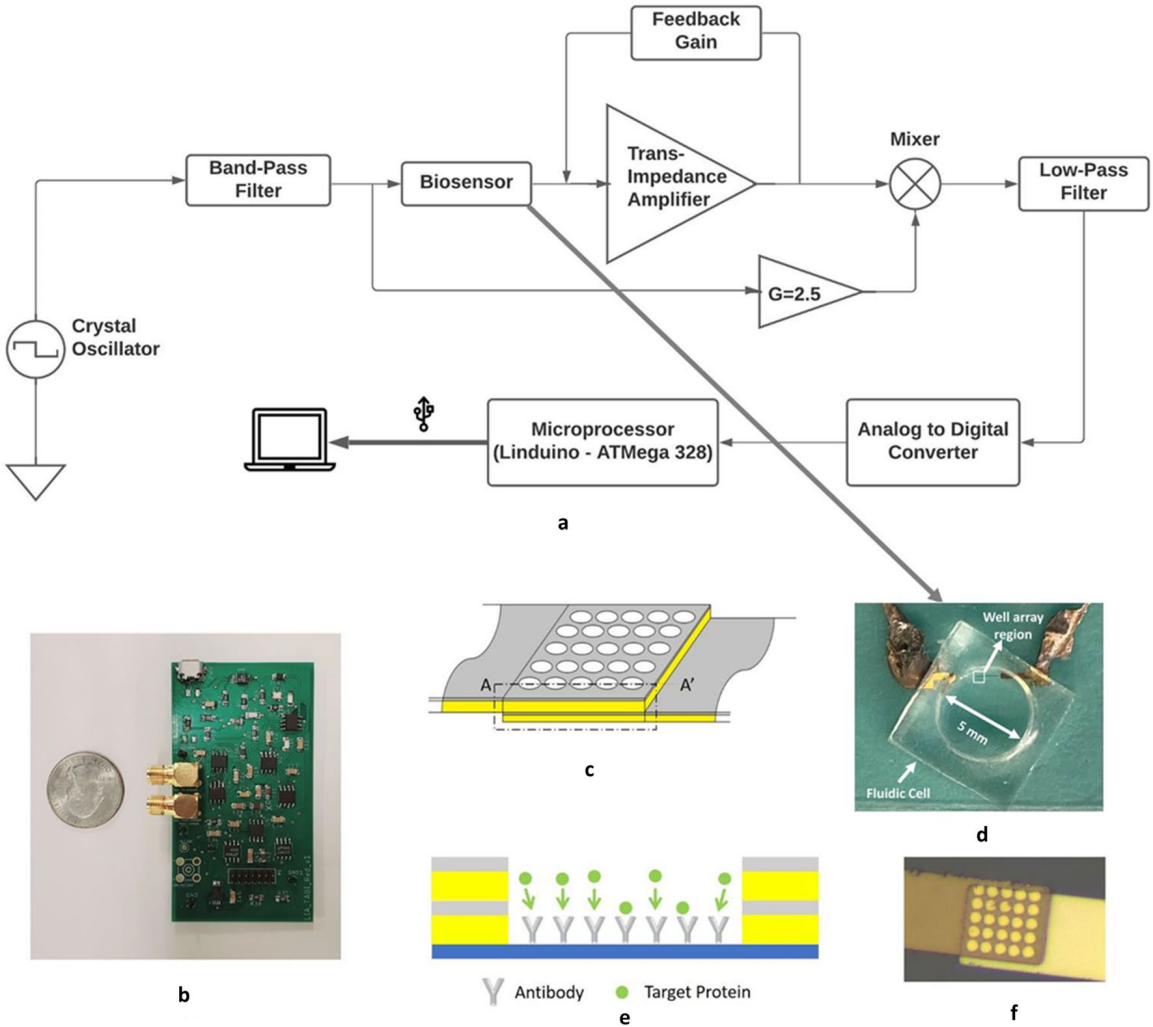
System Diagram of the Portable Electronic Readout System (**b**) Picture of the designed lock-in amplifier custom PCB including onboard ADC with small footprint (80 mm × 43.6 mm) (**c**) Schematic of the Nanowell Array Impedance Sensor (**d**) Picture of the nanowell array impedance sensor. (**e**) Principle of the nanowell array impedance sensor: the antibodies and target proteins occlude the current path and result in an increase in the impedance (**f**) Microscopic picture of the 5 × 5 nanowell array.

The basic architecture of the electronic readout system is presented in Fig. 1a. From our previous study using the nanowell array impedance sensor with the benchtop lock-in amplifier, we found the optimal operating frequency for operation to be 1 MHz and the optimal applied voltage to be 0.4 V for the detection of proteins^36^. A crystal oscillator provides the 1 MHz excitation signal for the biosensor. This is passed through an active bandpass filter to convert it to a sinusoidal signal of amplitude 400 mV. The bandpass filter is centered around 1 MHz and has a bandwidth of 100 kHz. We adjust the gain of the active bandpass filter such that we get a 0.4 V sinusoidal excitation signal at 1 MHz. This signal is then passed through the biosensor and fed to the mixer. The mixer mixes the excitation signal, which is amplified to 1 V prior to mixing, with the output signal from the biosensor. The mixed signal is subsequently passed through an active sixth order low-pass filter. This filter needs to have a low cut-off in order to reduce the noise of the system. In the case of the nanowell array impedance sensor, the signal we are trying to measure is very slow i.e., the adsorption of the probe molecules to the surface and the binding of the target molecules to these probe molecules. Both of these processes take considerable time usually in the order of minutes; therefore, we can choose the cut-off frequency for the low-pass filter as low as 10 Hz to reduce the overall noise of the system. Once we have removed the high frequency component of the mixed signal by low-pass filtering, we digitize the output using a 24-bit ADC. All of this circuitry including the ADC is onboard the 35cm^2^ custom PCB which can be seen in Fig. 1b. The ADC’s output is sent to a Linduino board (ATMega 328 Processor) connected to a laptop PC where the data are stored. The whole system is powered via USB ports on the PC.

### The nanowell array impedance sensor

The nanowell array impedance sensor’s design can be seen in Fig. 1c. There are two sets of overlapping electrodes each 100 nm thick that are separated by 40 nm Alumina (Al_2_O_3_). A microscopic image of the sensor can be seen in Fig. 1f. The electrodes are deposited on top of a glass substrate and are subsequently covered by another layer of Alumina (40 nm thick). 2 µm diameter wells (holes) are used to make space for the probe antibodies and the capture antigen. An ion current path would be formed between the two electrodes when the sensor submerged in the electrolyte environment and applied a driven voltage. The impedance of the sensor is measured in real time. Probe antibodies are physically absorbed in the micro-sized wells. The impedance across two electrodes increases with the target protein binding to the immobilized antibodies in the micro-wells as seen in Fig. 1e. The bound protein would block the conductive path between the top and bottom electrode. This results in the impedance increment. The amplitude of impedance increment is proportional to the change of output current, which is also determined by the concentration of target protein in samples. The sensor also benefits from the sample's high salt concentration. The higher salt concentration results in a larger current, corresponding to a more considerable change as the protein binding happens. The two electrodes extend to the bonding pads through the trace in the opposite direction, leaving the overlapping area of 20 µm x 20 µm, reducing the effects of parasitic capacitance. A 5 mm Polydimethylsiloxane (PDMS) well is bonded to the sensor surface to contain the fluid in the sensor area. A photograph of the nanowell array impedance sensor with the well bonded can be seen in Fig. 1d. The nanowells in the nanowell array impedance sensor serves the purpose of detecting and quantifying proteins in a given sample. The protein molecules are small and need a very sensitive sensing region for their detection. This region therefore needs to be small i.e. the order of nm to be sensitive enough to detect molecules as small as proteins. This sensitivity is provided by the nanowell senor array sensor’s gold electrode overlapping region where the nanowells are present. The distance between the two electrodes in this region is just 40 nm provided by the Alumina (Al_2_O_3_) layer between the two gold electrodes. Thus, when current is applied to these electrodes there is an electric field in the vicinity of these “walls” of the nanowells. This electric field exists between the two vertically spaced electrodes in this region. When this electric field is perturbed by the addition of antibodies and proteins, there is an impedance change. The impedance response of the sensor varies proportionally to the amount of protein present in the sensor. The exact impedance change depends on a number of parameters such as the double layer capacitance, the equivalent circuit model of the sensor, the solution resistance, and the binding kinematics of the antibody and the protein being used. Therefore, mathematically modeling these parameters is a comprehensive task and merits a study of its own. Our group has previously mathematically modeled these parameters and readers interested in such details are encouraged to refer to these works^38,39^. A self-built lock-in amplification system is used to measure the excitation current through the nanowell sensors. To maximize the sensitivity, it is extremely critical to perform the measurement in real-time, monitoring the impedance changes as the sensor's biological binding events occur.

### System characterization

**Figure 2:**
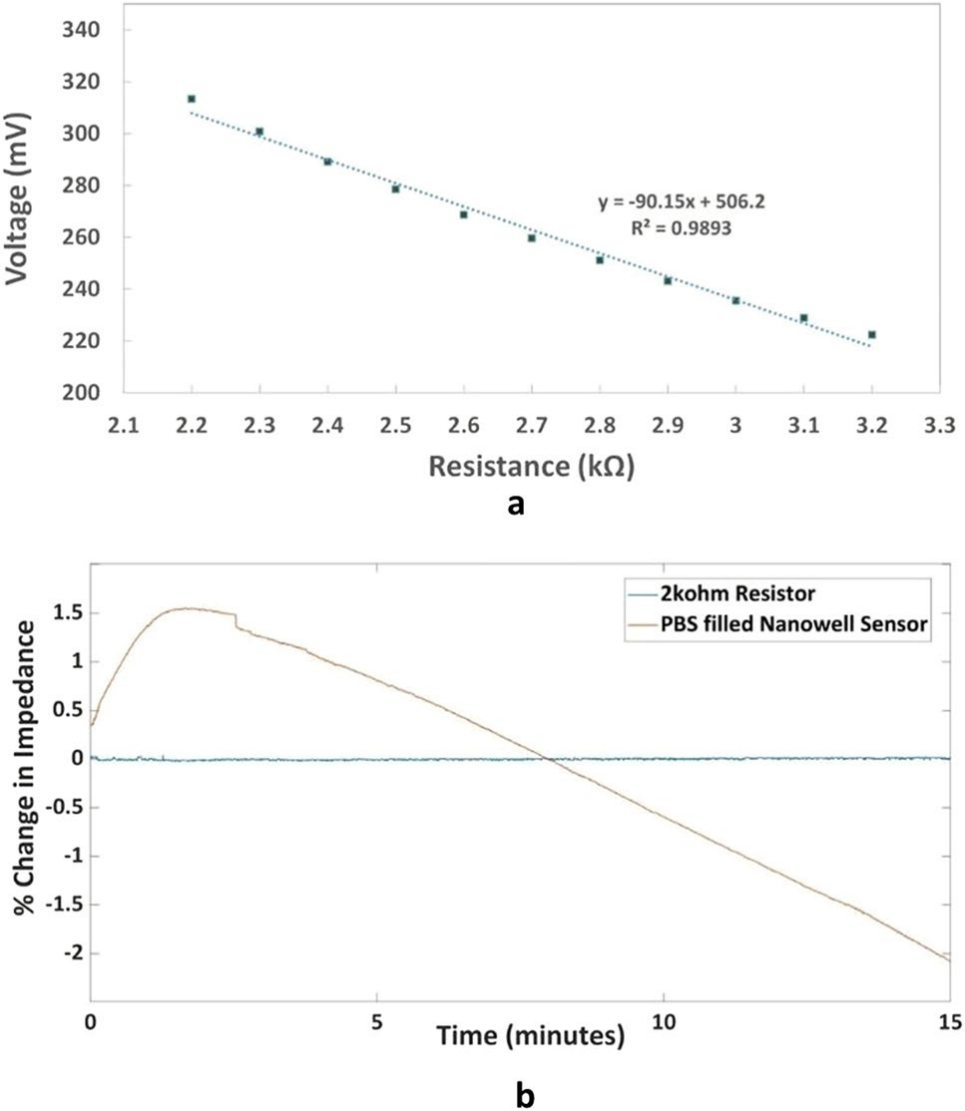
(**a**) Output Voltage (mV) at the Analog-to-Digital Converter (ADC) versus Resistance (kΩ) for the region of interest (**b**) Drift characterization of the circuit using a 2 kΩ resistor.

Since our sensor is used to detect proteins as a function of impedance, we want to characterize the performance of the system by using resistors. We found out through experimentation that the impedance of the nanowell array impedance sensor for detecting proteins lies around 2.7 kΩ. For this purpose, we recorded the output of the circuit by substituting resistors of various resistances in place of the sensor and calculating the mean output voltage. This information is summarized in the form a graph which can be seen in Fig. 2a. We perform a linear regression analysis in this region to determine the linearity. The R2 value in this region is 0.9893, indicating it is sufficiently linear for our application. The output voltage of our circuit drops approximately by 90 mV for a 1 kΩ change in resistance and it varies between 220 and 320 mV in the region of interest. Although the system is sufficiently linear for the purposes of our application, the mixer used in the system exhibits nonlinearity for low drive voltages. As a consequence, when our system is used with resistances greater than 4 kΩ it has a nonlinear response. These nonlinear characteristics of the AD835 mixer have been studied^40^ and will be addressed in a future version of this system to provide a linear response over a larger dynamic range. We tested the change in the output by varying the resistance with a change as small as 0.1% and were able to detect the difference in the output voltage. As an example, the average output voltage for a 3 kΩ resistor when used in place of the nanowell sensor was 235.5417 mV whereas for 3.003 kΩ, it is reduced to 235.0697 mV indicating a change of approximately 500 µV or 0.1% in the output voltage. An electronic drift in the baseline voltage is observed when the sensors are filled with the electrolyte solution. To make sure that this drift is a property common to the experiments with electrolyte filled sensors and not induced by the circuit itself, we performed an experiment where we observed the percentage change in the impedance over a period of 15 min when (a) a 2 kΩ resistor is used instead of the biosensor and (b) a sensor is filled with PBS. The drift characterization results can be seen in Fig. 2b. As can be seen from the Fig. 2b, the drift observed for the resistor is infinitesimally small compared to the sensor filled with PBS. Hence, we conclude that the circuit itself does not contribute significantly to the baseline drift.

### Noise analysis

**Figure 3:**
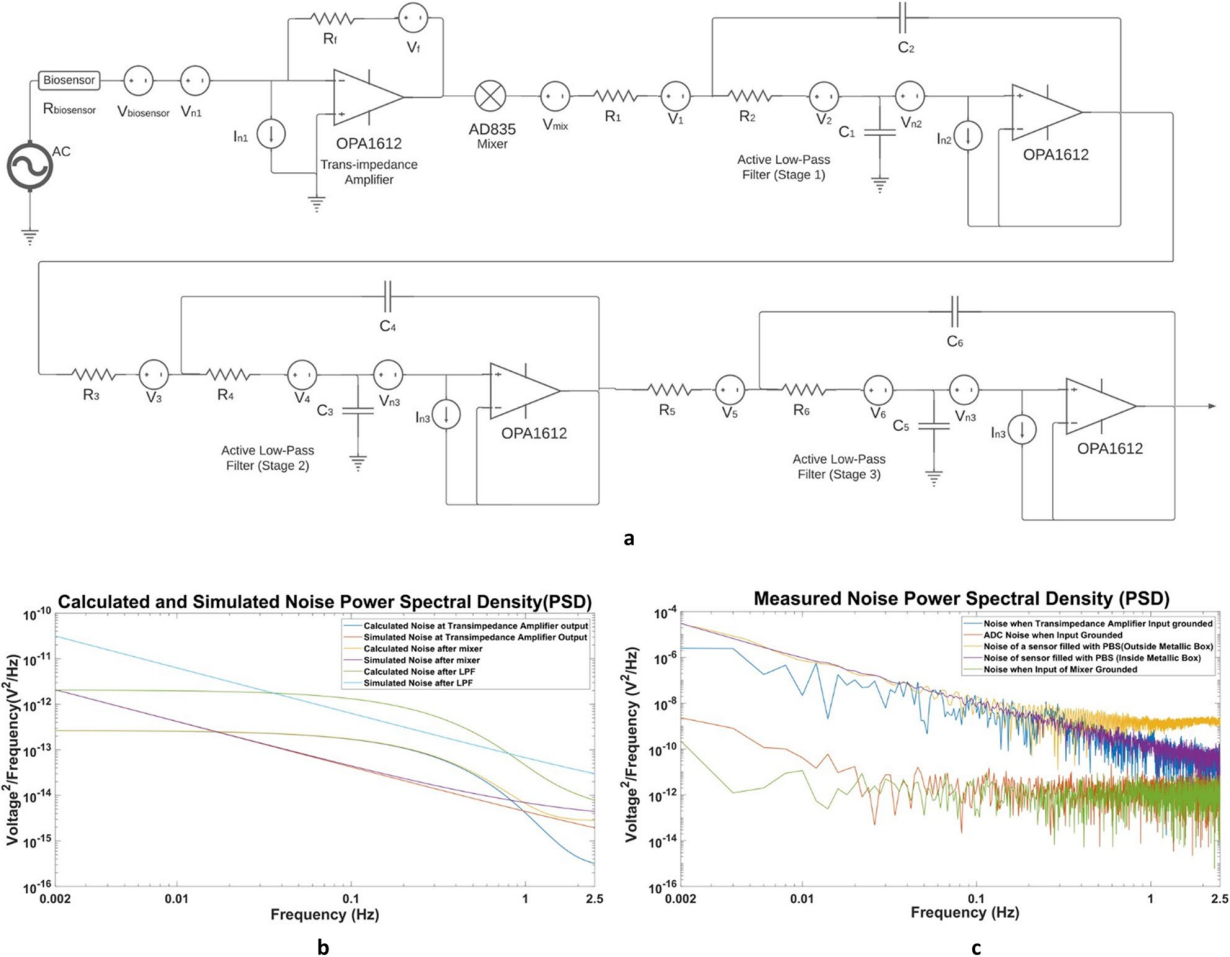
(**a**) Noise model of the lock-in amplifier (**b**) Calculated and Simulated Noise Power Spectral Density (**c**) Measured Noise Power Spectral Density under different conditions. Each case corresponds to a measurement time of 10 min.

Understanding the various noise contributors and minimizing the noise in the system is paramount for the detection of various biomarkers in biological matrices. We simulated the total noise of the system in LTSpice software and performed a comprehensive noise calculation by considering all the major sources of noise. The noise model is presented in Fig. 3a. We derived the equations for the noise of the system using the noise model presented in Fig. 3a. We calculated the noise of the system by using the output referred voltage noise of the system from the datasheet provided by the manufacturer. The part numbers for the components are illustrated in the Fig. 3a. We also compared this calculated noise with the simulated noise using LTSpice. This can be seen in Fig. 3b. Here, we present our derived equations for the noise of the system. The noise for the first stage which is the transimpedance amplifier is given by:

1 $$ {\text{E}}_{{{\text{n}}1}}^{2} = {\text{I}}_{{{\text{n}}1}}^{2} {\text{R}}_{{\text{f}}}^{2} + \left( {\frac{{{\text{R}}_{{\text{f}}}^{2} }}{{{\text{R}}_{{{\text{biosensor}}}}^{2} }}} \right)\left( {V_{biosensor}^{2} + V_{n1}^{2} } \right) + V_{f}^{2} $$

The noise for the mixer stage is:

2 $$ E_{n2}^{2} = V_{mix}^{2} + E_{n1}^{2} $$

The output referred voltage noise of the first stage of the active low-pass filter is given by:

3 $$ {\text{E}}_{{{\text{n3}}}}^{{2}} { = }\frac{{1}}{{{\text{(R}}_{{1}} {\text{R}}_{{2}} {\text{ + (R}}_{{1}} {\text{ + R}}_{{2}} {\text{)Z}}_{{{\text{C2}}}} {\text{ + Z}}_{{{\text{C1}}}} {\text{Z}}_{{{\text{C2}}}} )^{2} }}{\text{[Z}}_{{{\text{C1}}}} {\text{Z}}_{{{\text{C2}}}} {\text{(V}}_{{1}} {\text{ + E}}_{{{\text{n2}}}} {\text{) + (Z}}_{{{\text{C1}}}} {\text{Z}}_{{{\text{C2}}}} {\text{ + R}}_{{1}} {\text{Z}}_{{{\text{C1}}}} {\text{)V}}_{{2}} {\text{ + (R}}_{{1}} {\text{R}}_{{2}} {\text{ + (R}}_{{1}} {\text{ + 2R}}_{{2}} {\text{)Z}}_{{{\text{C2}}}} {\text{ + Z}}_{{{\text{C1}}}} {\text{Z}}_{{{\text{C2}}}} {\text{)V}}_{{{\text{n2}}}} {]}^{{2}} { + }\left( {\frac{{{\text{R}}_{{1}} {\text{Z}}_{{{\text{C1}}}} }}{{{\text{(R}}_{{1}} {\text{ + R}}_{{2}} {\text{ + Z}}_{{{\text{C1}}}} }}} \right)^{{2}} {\text{I}}_{{{\text{n2}}}}^{{2}} $$

Similarly, the noise for the second stage of the active low-pass filter is:

4 $$ {\text{E}}_{{{\text{n4}}}}^{{2}} = \frac{{1}}{{{\text{(R}}_{{3}} {\text{R}}_{{4}} {\text{ + (R}}_{{3}} {\text{ + R}}_{{4}} {\text{)Z}}_{{{\text{C4}}}} {\text{ + Z}}_{{{\text{C3}}}} {\text{Z}}_{{{\text{C4}}}} {)}^{{2}} }}{\text{[Z}}_{{{\text{C3}}}} {\text{Z}}_{{{\text{C4}}}} {\text{(V}}_{{3}} {\text{ + E}}_{{{\text{n3}}}} {\text{) + (Z}}_{{{\text{C3}}}} {\text{Z}}_{{{\text{C4}}}} {\text{ + R}}_{{3}} {\text{Z}}_{{{\text{C3}}}} {\text{)V}}_{{4}} {\text{ + (R}}_{{3}} {\text{R}}_{{4}} {\text{ + (R}}_{{3}} {\text{ + 2R}}_{{4}} {\text{)Z}}_{{{\text{C4}}}} {\text{ + Z}}_{{{\text{C3}}}} {\text{Z}}_{{{\text{C4}}}} {\text{)V}}_{{{\text{n3}}}} {]}^{{2}} { + }\left( {\frac{{{\text{R}}_{{4}} {\text{Z}}_{{{\text{C3}}}} }}{{{\text{(R}}_{{3}} {\text{ + R}}_{{4}} {\text{ + Z}}_{{{\text{C3}}}} }}} \right)^{{2}} {\text{I}}_{{{\text{n3}}}}^{{2}} $$

Finally, the noise for the third stage of the active low-pass filter is:

5 $$ {\text{E}}_{{{\text{n5}}}}^{{2}}  = \frac{{1}}{{{\text{(R}}_{{5}} {\text{R}}_{{6}} {\text{ + (R}}_{{5}} {\text{ + R}}_{{6}} {\text{)Z}}_{{{\text{C6}}}} {\text{ + Z}}_{{{\text{C5}}}} {\text{Z}}_{{{\text{C6}}}} {)}^{{2}} }}{\text{[Z}}_{{{\text{C5}}}} {\text{Z}}_{{{\text{C6}}}} {\text{(V}}_{{5}} {\text{ + E}}_{{{\text{n4}}}} {\text{) + (Z}}_{{{\text{C5}}}} {\text{Z}}_{{{\text{C6}}}} {\text{ + R}}_{{5}} {\text{Z}}_{{{\text{C5}}}} {\text{)V}}_{{6}} {\text{ + (R}}_{{5}} {\text{R}}_{{6}} {\text{ + (R}}_{{5}} {\text{ + 2R}}_{{6}} {\text{)Z}}_{{{\text{C6}}}} {\text{ + Z}}_{{{\text{C5}}}} {\text{Z}}_{{{\text{C6}}}} {\text{)V}}_{{{\text{n4}}}} {]}^{{2}} { + }\left( {\frac{{{\text{R}}_{{6}} {\text{Z}}_{{{\text{C5}}}} }}{{{\text{(R}}_{{5}} {\text{ + R}}_{{6}} {\text{ + Z}}_{{{\text{C5}}}} }}} \right)^{{2}} {\text{I}}_{{{\text{n4}}}}^{{2}} $$

The transimpedance amplifier noise is the most significant and it affects all of the subsequent stages. The calculated and simulated noise spectrum for the system can be seen in Fig. 3b. Further investigation reveals that the In_1_^2^R_f_^2^ term in (1) is the dominant noise component. Hence, the total noise of the system highly depends on the feedback resistance. We find out that the total RMS noise of the system to be approximately 678nV from the simulations whereas the total RMS noise estimated from our noise calculation is 702 nV.

### Noise measurements

We measured the noise of the system under different conditions. The measurements are summarized in Fig. 3c. The noise of the ADC when its input is grounded is the lowest. The Total ADC RMS noise is approximately 8.45 µV. We also measured the noise of the circuit by connecting a 3.6 kΩ resistor instead of the biosensor and grounding the input of the transimpedance amplifier. We then grounded the input of the mixer and measured the noise again. We replaced the resistor with a sensor and filled it with PBS to measure the noise. By experimentation, we found that the output noise voltage observed by replacing the resistor with a sensor filled with PBS is similar to that of a 3.6 kΩ resistor. Hence, we used 3.6 kΩ as the value of the Rbiosensor in our simulations and calculations. The measured noise RMS value is approximately167.45 µV when the system is placed inside a metal box. The experiments were performed by placing the circuit and sensor inside a metal box used as a Faraday cage for reducing noise but doing so did not result in a significant reduction in the noise. The measured output voltage noise is significantly higher than our calculations and simulations since the noise of the voltage regulators and the power supply circuit dominate the noise generated by the amplifier and resistors. Our noise calculations and simulations do not take these additional sources of noise into consideration such as the noise added due to linear Low-Dropout Voltage Regulators providing + 3.3 V and −3.3 V, the frequency generating circuit consisting of a crystal and a Band-pass Filter, and the interference due to the environmental noise present in the laboratory setting.

## Results

### Experimental setup

The experimental setup using the electronic readout system with the nanowell array impedance sensor is shown in Fig. 6b. All the experiments were performed inside the metal box with the lid closed and opened only for pipetting the reagents and samples into the nanowell array impedance sensor. The whole system is powered via USB ports of the laptop which are connected to regulator circuits providing + 3 V and −3 V for the custom PCB. The PCB contains the regulator circuits, frequency generating circuit, the lock-in amplification, and the 24-bit Analog-to-Digital converter. Jumper wires are used to connect the nanowell array impedance sensor to the custom PCB. A Coaxial interface is also available on the PCB for connecting the PCB to the sensor. The data are digitized by using the 24-bit delta-sigma Analog-to-Digital Converter (ADC) which is connected to a ATMega 328 Processor using Serial Peripheral Interface (SPI). The data is transferred to the laptop computer using SPI where it is monitored in real-time and stored. We performed 2 sets of experiments to verify the operation of our system. Firstly, we determined if we could detect the adsorption of antibodies at various concentrations to the sensor surface. Secondly, we performed an experiment to detect 45 pM protein (IL-6). We repeated this experiment 3 times. A detailed explanation for these experiments is presented in the subsequent sections.

### Antibody adsorption quantification

**Figure 4:**
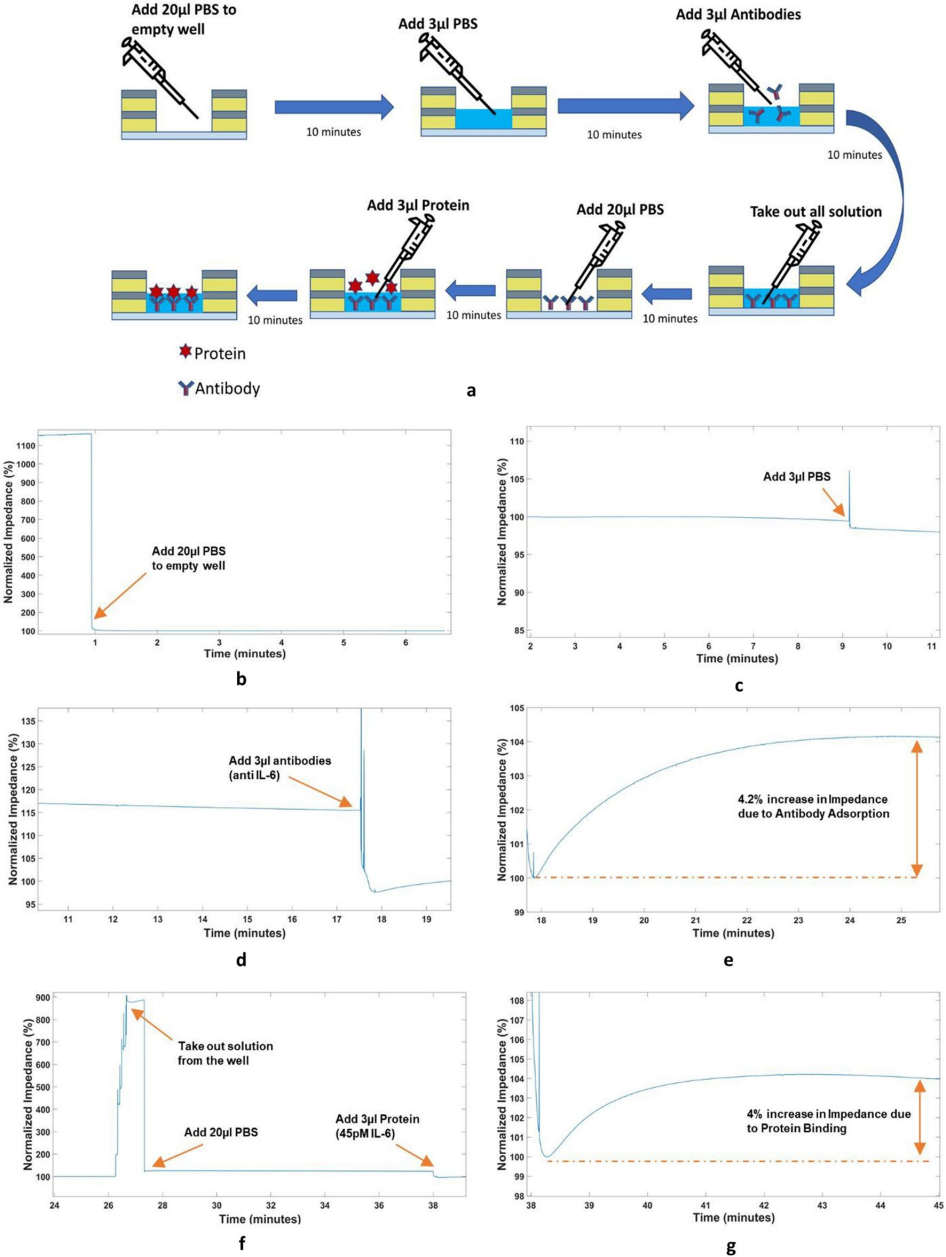
Protein Detection Experiment (**a**) Process Diagram for the experimental protocol used (**b**) Adding 20 μl PBS to an empty nanowell sensor (**c**) Adding 3 μl PBS to the sensor (**d**) Adding antibodies to the sensor (**e**) Approximately 4.2% increase in impedance due to antibody adsorption (**f**) Clearing the nanowell sensor by pipetting out solution and adding 20 μl PBS before adding 3 μl of 45 pM protein (**g**) Approximately 4% change in impedance observed due to protein binding.

Monoclonal Human/Primate IL-6 Antibody (MAB206, R&D Systems) was used for these experiments. 3 µl aliquots of the antibody solution at different concentrations were prepared by diluting the antibody in 1 × PBS. We prepared 7 different concentrations of antibodies: 1 µg/ml, 5 µg/ml, 10 µg/ml, 20 µg/ml, 30 µg/ml, 50 µg/ml, and 100 µg/ml. The protocol of the experiments is described here. We start the experiment by first adding 20 µl PBS to the empty nanowell sensor. We then wait for 10 min. Next, we add 3 µl PBS as a negative control. This step is performed to ensure that the change we observe when we add antibodies is different from the change due to simply adding PBS. We wait for another 10 min. After waiting, we proceed to add our antibodies to the sensor. We add 3 µl of the prepared antibody concentration aliquot to the solution. Through experimentation, we know that the shape of the data obtained after this step needs to be identical to that shown in Fig. 4e. As can be seen in Fig. 4d, there is an initial abrupt baseline shift due to adding the antibody solution and the impedance increases subsequently in an exponential fashion for a period of roughly 7–10 min after the initial baseline shift. The data obtained from the experiments are observed in real-time to ensure standard operation. These data are analyzed using MATLAB to find out the percent impedance change. The results of these antibody adsorption experiments are summarized in Fig. 6a. The impedance correlates with the antibody concentration between 1 µg/ml and 100 µg/ml. There is an almost flat response above 50 µg/ml, after which the impedance change does not increase. This is due to the fact that the sensor has saturated and there is no more room for antibodies to be adsorbed to the surface of the sensor’s active regionThe dotted line in Fig. 6a presents the saturation of the nanowell array impedance sensor, whereas the linear region of the curve is shown in Fig. 6a using a straight solid line. A linear regression analysis is performed to determine the linearity of the observed change in impedance to the antibody concentration in the linear region. The r^2^ value from the linear regression analysis for the linear region comes out to be 0.9158. The antibody adsorption experiments are useful to indicate the presence of the antibody and providing a rough estimate of the concentration. However, for a more accurate result for antibody concentration, it is recommended that the sensor surface be pre-treated with primary antibodies and/or antigens specific to the target detection molecule and then the impedance be measured. This protocol has been demonstrated for detecting IL-6 protein at 45 pM, which is presented in the next section.

### Protein detection

**Figure 5:**
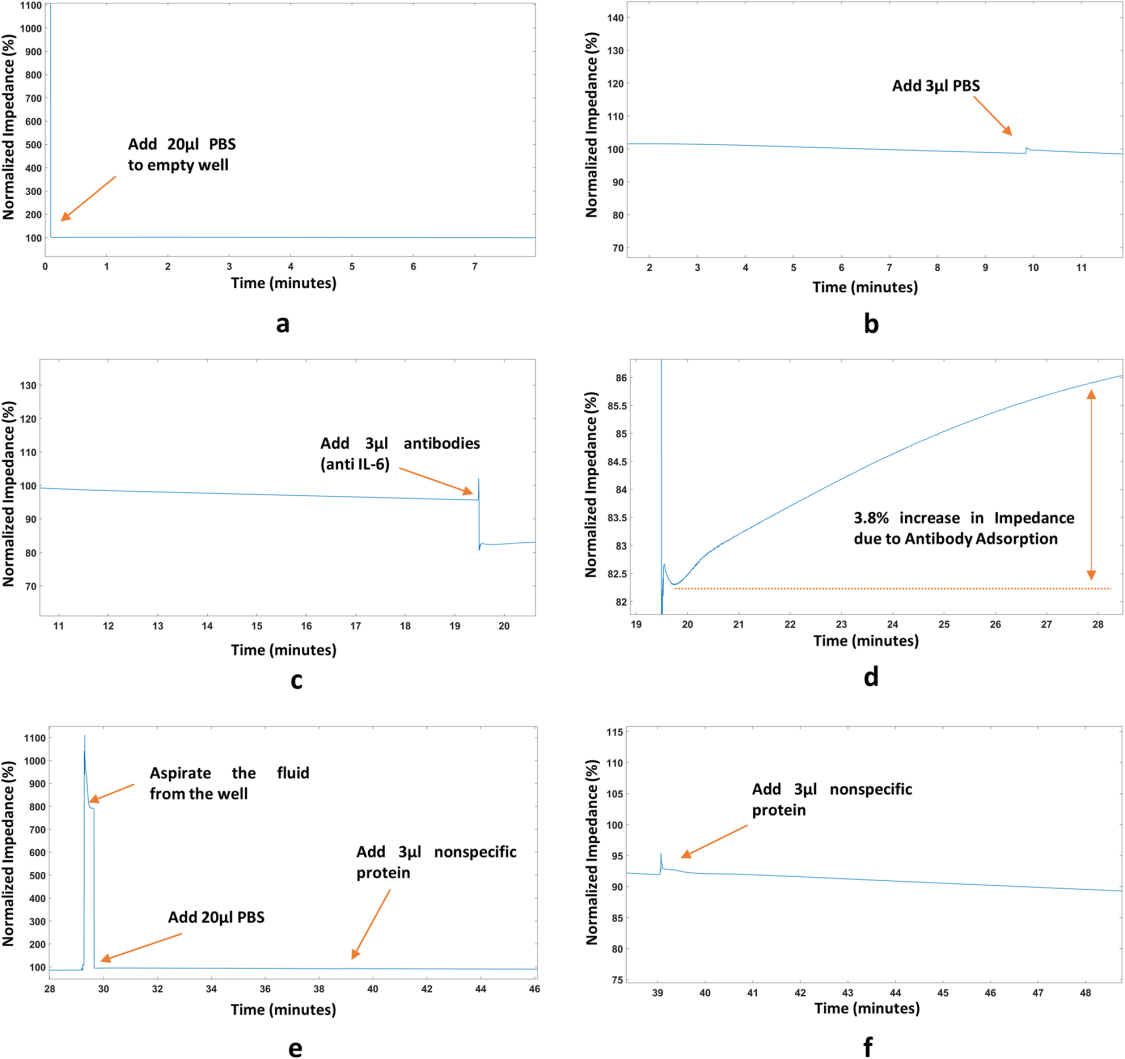
Nonspecific protein experiment (Negative Control) (**a**) Adding 20 μl PBS to an empty nanowell sensor (**b**) Adding 3 μl PBS to the sensor (**c**) Adding antibodies (anti IL-6) to the sensor (**d**) Approximately 3.8% increase in impedance due to antibody adsorption (**e**) Clearing the nanowell sensor by pipetting out solution and adding 20 μl PBS before adding 3 μl of Recombinant Human SARS-CoV-2 Spike Glycoprotein S1 (ab272105, Abcam) (**f**) Approximately -1.3% change in impedance observed. This is similar to adding PBS to the well.

**Figure 6:**
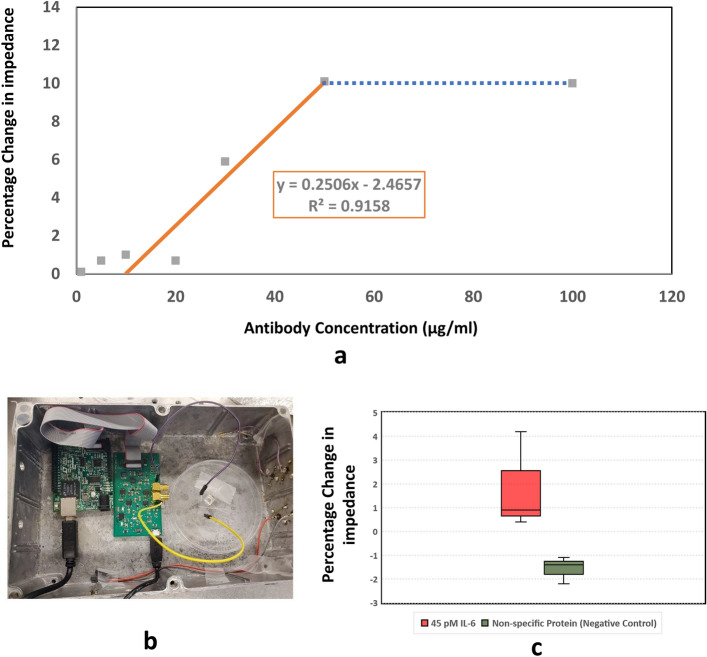
(**a**) Percentage Change in Impedance observed for the antibody adsorption experiments with different antibody concentrations. (**b**) Experimental Setup. From left to right: Controller board (Linduino- ATMega328 Processor), Custom PCB including frequency generation, lock-in amplification, and the 24-bit Analog-to-Digital Converter, Nanowell Array Impedance sensor on a petri dish. The experiments were performed with the metal box (acts as Faraday cage) with lid closed to reduce interference (**c**) Box plot of the experiment for protein detection in PBS. The experiments were repeated 3 times with 45 pM IL-6 and with non-specific protein i.e. SARS CoV-2 glycoprotein.

We verified the electronic readout system by detecting protein detection. The protocol for the experiment can be seen in Fig. 4a. We repeated this experiment 3 times. The detailed protocol of the experiment is presented here. We start by adding 20 µl 1 × PBS to an empty nanowell sensor. We should see an abrupt decrease in the impedance since the PBS filled sensor completes the circuit. This step can be seen in Fig. 4b. We then wait for the response to stabilize. This step takes about 10 min. We add another 3 µl volume of PBS to the nanowell sensor. This step can be seen in Fig. 4c. The change in impedance should be small in this step compared to when we add antibodies and subsequently, proteins. Then we wait for the response to stabilize. This step also takes approximately 7–10 min. Next, we proceed to add antibodies to the sensor. We add 3 µl antibody solution to the nanowell sensor in this step. We should see an exponential increase in the impedance after an initial baseline shift as can be seen in Fig. 4d,e due to the adsorption of these antibodies to the sensor’s surface occluding the current path. We wait for about 10 min for the antibodies to be adsorbed to the surface before taking out the extra solution and again adding 20 µl PBS. This step is illustrated in Fig. 4f. We again wait for about 10 more minutes before the response is stabilized. The final step is to add 3 µl of the desired protein solution for detection. For this set of experiments, we used 45 pM Recombinant Human IL-6 Protein (206-IL-010, R&D Systems). The shape of the output voltage for this step should be identical to the step where we add antibodies, which can be seen in Fig. 4g since the binding of the proteins to the antibodies should occlude the current path and result in an increase in the impedance. Although we have followed an experimental protocol that takes about 45 min, the experiment can be carried out in as little as 10 min in a real world setting by skipping the validation steps for adding PBS and directly adding the protein to the functionalized sensor surface. We have used Monoclonal Human/Primate IL-6 Antibody (MAB206, R&D Systems) with the Recombinant Human coronavirus SARS-CoV-2 Spike Glycoprotein S1 (ab272105, Abcam) as a negative control. The experimental protocol and the step-by-step result for the negative control experiment is presented in Fig. 5. Both the protein quantification and the negative control experiments were repeated 3 times. The results for all 3 experiments are summarized in the form of a boxplot shown in Fig. 6c.

We performed statistical analysis of the data using R software. The mean for the percentage change in impedance due to negative control is − 1.56 and for the percentage change in impedance due to binding of proteins is 1.81. Shapiro–Wilk tests for the negative control yields values (W = 0.93557, *p*-value = 0.5098) and for the protein binding yields (W = 0.8675, *p*-value = 0.2884) indicating that the negative control and the specific protein data does not deviate significantly from a normal distribution. We also perform Bartlett’s K-squared test to determine homogeneity of variances. We get the values (Bartlett's K-squared = 2.1425, df = 1, *p*-value = 0.1433) indicating that we cannot reject the null hypothesis and thus points towards homoscedasticity of the data. The variances of the protein experiments are 4.3833 and the variance of the negative control is 0.32333. The ratio between these variances is therefore 13.185. We also perform F-test to determine if the variances are statistically significant. The F-test yields the values (Fcalc = 13.186, Fcritical = 0.0526, *p*-value = 0.141). Since Fcalc > Fcritcal, this indicates that the data comes from populations with statistically significant variances.

## Conclusion

We present the design of a portable, low noise electronic readout system to be used in conjunction with nanowell array impedance sensors for label-free sensing of proteins. The designed system has a small footprint of 35cm^2^. We carried out a comprehensive noise analysis of the system by calculating and simulating the output voltage referred noise and comparing it with the actual noise exhibited by the system. We have demonstrated the ability of the system to quantify antibodies by using varying antibody concentrations. We also determined the utility of the system to detect proteins by repeated detection of 45 pM IL-6 three times. Although we used a relatively slow protocol for our experiments to ensure robustness, our system has the ability to perform protein detection with a sample-to-answer turnaround time of 10 min when used in a practical setting. Although this work highlights the application of this system to be used for protein detection, it can be potentially used to study and measure the binding kinetics of the antibodies and proteins. To achieve this using the current system, we need only make slight modifications to the experimental protocol and analysis of the data. We have used a laptop computer and a separate board for the ATMega328 (Linduino) for storing the data. This can be replaced with an onboard microprocessor and the result can be transmitted wirelessly to a smartphone via Bluetooth in a future version of the system as we have demonstrated previously for a portable, microfluidic impedance cytometer^41^. A 3D printed package for the device with compartments for the circuit, the microcontroller, and the sensor is also a potential area where improvement can be made for using the system at Point-of-Care.

## Supplementary Information

Supplementary Information.

## Data Availability

The data underlying this article will be shared upon reasonable request to the corresponding author.
