# Supplementary material for: A portable analog front-end system for label-free sensing of proteins using nanowell array impedance sensors
Source: Sci Rep. 2022 Nov 22;12:20119. doi: 10.1038/s41598-022-23286-7 (PMC9684124; doi:10.1038/s41598-022-23286-7)
Supplement: Supplementary file 1 — Supplementary Information. [file 41598_2022_23286_MOESM1_ESM.docx]

Table S1: A head to head comparison of the commercial lock-in amplifier and the proposed custom lock-in amplification system

|  | **Commercial lock-in amplifier (Zurich Instruments, HF2IS)** | **Proposed system** |
| --- | --- | --- |
| **Protein detection at picomolar levels** | **✓** | **✓** |
| **Multiple excitation frequencies** | **✓** | **🗶** |
| **Multiple excitation voltages** | **✓** | **🗶** |
| **Adaptive Filters** | **✓** | **🗶** |
| **Portable** | **🗶** | **✓** |
| **Inexpensive** | **🗶** | **✓** |
